# Overexpression of GDNF in Spinal Cord Attenuates Morphine Analgesic Tolerance in Rats with Bone Cancer Pain

**DOI:** 10.3390/brainsci12091188

**Published:** 2022-09-03

**Authors:** Wei Xu, Zhuofeng Ding, Zongbin Song, Jian Wang, Jie Zhang, Wangyuan Zou

**Affiliations:** 1 Department of Anesthesiology, The Maternal and Child Health Hospital of Hunan Province, Changsha 410010, China; 2 Department of Anesthesiology, Xiangya Hospital, Central South University, Changsha 410008, China

**Keywords:** bone cancer pain, morphine tolerance, glial cell line-derived neurotrophic factor, MOR, spinal

## Abstract

Bone cancer pain (BCP) is one of the typical and distressing symptoms in cancer patients. Morphine is a widely used analgesic drug for BCP; however, long-term morphine administration will lead to analgesic tolerance. Our previous study indicated that spinal glial cell line-derived neurotrophic factor (GDNF) exerts analgesic effects in rats with BCP. In this study, BCP was established by inoculated Walker 256 carcinoma cells into rat tibias, while morphine tolerance (MT) was induced by intrathecally injecting morphine twice daily from the 9th to 15th postoperative day (POD) in BCP rats. The BCP rats developed mechanical and thermal hyperalgesia on POD 5 and it lasted to POD 15. The analgesic effect of morphine was decreased after repeat administration. Western blots and immunochemistry tests showed that GDNF was gradually decreased in the spinal cord after the development of MT in rats with BCP, and GDNF was colocalized with the μ opioid receptor (MOR) in the superficial laminate of the spinal cords. The overexpression of GDNF by lentivirus significantly attenuated MT, and restored the expression of MOR in the spinal cord. In summary, our results suggest that the reduction of GDNF expression participated in the development of MT in rats with BCP and could be a promising therapeutic option for BCP.

## 1. Introduction

Bone cancer pain (BCP) is induced by bone cancer or secondary bone metastasis in other cancer, including breast, lung, and prostate cancer, and is highly difficult to manage [1,2]. Morphine has always been the most suggested drug for alleviating moderate to severe cancer pain [3,4]. However, the repeated administration of morphine may lead to various adverse effects, such as nausea, constipation, itching, and tolerance [5]. Morphine tolerance (MT) is defined as the reduction of analgesic efficacy of morphine after prolonged usage and requiring larger doses for the maintenance of analgesia. The mechanisms underlying MT are very complex and have not been fully elucidated so far. Furthermore, there is heterogeneity in the analgesic effect of opioids on different pain types. Morphine exerts its analgesic function mainly by targeting μ opioid receptors (MOR) [6,7]. Our previous studies found that the expression of MOR decreased in the lumbar spinal cord of BCP rats [8,9]. Thus, it is important to explore the mechanism of MT under the condition of BCP.

Neurotrophic factors play an important role in the survival, growth, and differentiation of distinct populations of neurons. More recently, neurotrophic factors have been found to be implicated in many forms of neuroplasticity. Glial cell line-derived neurotrophic factor (GDNF) belongs to the neurotrophic factor family and was first shown to protect and maintain the development of brain dopaminergic neurons [10]. Recently, evidence has shown that GDNF could participate in the development of opioid addiction and tolerance. The injection of GDNF into the specific brain regions within the ventral tegmental area (VTA) alters neurobiochemical adaptations and behavioral changes to repeated cocaine or morphine treatment. In contrast, the intra-VTA injection of the anti-GDNF antibody enhanced the responses to cocaine treatment [11]. The repeated administration of morphine leads to an enhancement in response to the morphine induced locomotor-stimulating effect, and to up-regulation of the GDNF expression in the nucleus accumbens [12]. Our previous study showed that spinal GDNF was downregulated after rats developed BCP, and the overexpression of GDNF could alleviate bone cancer pain, which suggests that spinal GDNF acts as an important regulator in the development of BCP [13]. However, the role of spinal GDNF in the development of MT with BCP has not been fully studied yet. Thus, this study was designed to explore whether spinal GDNF participates in the development of MT with BCP and the possible mechanism of GDNF in regulating MT.

## 2. Materials and Methods

### 2.1. Animals

Adult female Sprague-Dawley rats (180–220 g) were provided by the Hunan SJA Laboratory Animal Co. All of the animals were housed in independent cages in a temperature-controlled environment at 22 °C with free access to food and water. All animal experimental procedures followed the guidelines of the ethics committee of the International Association for the Study of Pain and were approved by the Central South University Institutional Animal Care and Use Committee. In total, 108 rats were used, and no rats were was lost due to complications during the experiment. There were eight rats per group for the behavioral test. After the behavioral test was completed, three rats per group were analyzed for immunohistochemistry, and four rats per group were analyzed for Western blotting.

### 2.2. Intrathecal Catheterization

The animals were anesthetized with isoflurane to implant intrathecal catheters for drug delivery, as previously described [14]. Briefly, a sterilized polyethylene-10 catheter filled with normal saline was inserted into the subarachnoid space of the L4-L5 spinal segmental level. The end of the intrathecal catheter was buried under the skin, and the tip of the tubing was tunneled subcutaneously to the neck and fixed appropriately. Rats with paralysis or motor weakness were excluded from the study.

### 2.3. Induction of Bone Cancer Pain and Morphine Tolerance Model

The model of BCP due to metastasized breast cancer was constructed as previously described [13]. In brief, Walker 256 mammary gland cells were slowly inoculated into the intramedullary space of the left tibia of rats. Walker 256 breast carcinoma cells were purchased from the Beijing Dingguo Changsheng Biotechnology Co. The carcinoma cells were washed with sterilized PBS and adjusted to an appropriate concentration (4 × 10^5^/ μL). A volume of 10 μL of cell suspension was injected into the tibial medullary cavity. In parallel, sham-operated rats received heat-inactivated Walker 256 cancer cells instead. The bone destruction of carcinoma cells was assessed by X-ray and macroscopic observation. Radiographs were taken from the ipsilateral tibia of BCP rats on postoperative day (POD) 1, POD 9, and POD 16. On POD16, BCP rats were deeply anesthetized with isoflurane and transcardially perfused with saline, followed by 4% paraformaldehyde, and both the left and right tibial bone were removed to visualize the degree of bone destruction.

In order to induce MT in BCP rats, from POD 9 to POD 15 of BCP, morphine (10 μg, 10 μL) or saline (10 μL) was repeated intrathecally administered twice daily (08:00 a.m./ 16:00 p.m.) for 7 consecutive days.

### 2.4. Behavioral Test

#### 2.4.1. Mechanical Paw Withdrawal Threshold (PWT) Test

Mechanical hyperalgesia was evaluated using von Frey filaments, as previously described [13]. The rats were habituated to the environment for 30 min before testing. Von Frey filaments with different forces were vertically applied to the middle of the plantar surface of rats for 3 s. Licking or withdrawal of the left paw was defined as a positive reaction. The lowest filament that elicited a positive response from the paw was recorded as the PWT. Each test was repeated three times with an interval of at least 5 min.

#### 2.4.2. Paw Withdrawal Latency (PWL) Test

Thermal hyperalgesia was determined by the left paw withdrawal threshold in response to radiant thermal stimulation with a Hargreaves apparatus (Ugo Basile, Comerio, Italy), as previously described [14]. In brief, the experimental rats were placed on the glass platform and acclimated for 15 min. A light beam of the radiated thermal stimulus was focused on the middle of the plantar surface of the left hind paw. Withdrawal or licking of the left hind paw was recorded as a positive response to the thermal stimuli. To avoid hind paw damage, the cut-off time to radiant heat was set to 30 s, and the intensity was set to 65.

#### 2.4.3. Tail-Flick Latency (TF) Test

A tail-flick test was employed to assess the antinociception of morphine, as previously described [15]. The distal 1/3 of the tail was placed above the heat source. The cut-off time to radiant heat was set to 20 s, the intensity was set to 54 to prevent potential damage to the tail. The TF latencies were expressed as the percentage of the maximum possible effect (%MPE) to evaluate the antinociception of morphine. %MPE = 100 × (post drug latency-baseline latency)/(20-baseline latency).

### 2.5. Lentivirus Delivery

The lentiviral vector (GV287-GFP) containing the full-length GDNF coding sequences (LV-GDNF) and negative control sequence (LV-NC) were purchased from GENECHEM (GENECHEM, China). The titer of lentivirus used for injection was 1 × 10^9^ TU·mL^−1^. The lentivirus mediated GDNF or LV-NC was intrathecally injected with a volume of 10 μL on POD 7 after tumor cell inoculation.

### 2.6. Immunohistochemistry

The rats were deeply anesthetized with isoflurane and transcardially perfused with normal saline and 4% paraformaldehyde in PBS (pH 7.4). After perfusion, the lumbar spinal cord fragments were carefully dissected out and post-fixed at 4 °C for 4 h, followed by cryoprotection in 15% sucrose in PBS at 4 °C overnight. Then, the sample of the spinal cord were transferred to 30% sucrose for 3 days. Transverse spinal sections (10 μm) were cut in a cryostat (Leica Microsystems, Wetzlar, Germany). The sections of spinal cord were blocked with 10% normal donkey serum and 0.1% Triton X-100 in PBS for 1 h at 24 °C and then treated with rabbit anti-GDNF polyclonal antibody (1:100, Santa Cruz), mouse anti-MOR antibody (1:100, Abcam), and incubated at 4 °C in the refrigerator overnight. After washing with PBS, the sections of the spinal cord were incubated with appropriated secondary anti-mouse or rabbit antibodies labeled with Alexa Fluor 488 (1:400; Jackson) or Alexa Fluor 594 (1:400; Jackson) at 24 °C for 2 h. Fluorescence images of the spinal cords were captured with a Leica TCS SP5 confocal laser scanning microscope (Leica Microsystems).

### 2.7. Western Blotting

The rats were deeply anesthetized and sacrificed by decapitation after the behavioral tests. The L4-6 spinal cord tissues were quickly dissected and homogenized in a cold RIPA lysis buffer and then centrifuged for 5 min at 14,000× *g* at 4 °C. The supernatant of the sample was collected and the protein concentration was quantified. Following the addition of a 5 × sample buffer, the samples of the spinal cord were heat-denatured at 100 °C for 5 min. Fifty micrograms of protein per sample were resolved by 10% sodium dodecyl sulfate-polyacrylamide gel electrophoresis (SDS-PAGE) and transferred to nitrocellulose membranes. The membranes were then blocked with 5% non-fat milk for 2 h at 24 °C. Thereafter, the membranes were incubated with a specific primary antibody for rabbit anti-GDNF (1:400, Abcam), mouse anti-MOR (1:600, Abcam), and rabbit anti-GAPDH overnight at 4 °C. The next day, the membranes were incubated with the corresponding rabbit, or mouse Horseradish Peroxidase (HRP)-conjugated secondary IgG (1:20,000, Jackson ImmunoResearch, West Grove, PA, USA) for 1 h at 24 °C. Reactive bands were visualized with the enhanced chemoluminescent detection method with a digital camera system (cool snap HQ2; Photometrics^®^, Tuscon, AZ, USA) and were semi-quantitatively analyzed with a relative density value similar to that for the GAPDH.

### 2.8. Statistical Analyses

Graphpad Prism 9.0 was used to conduct all of the statistical analyses. Measurement data were presented as mean ± SD. Data from behavioral tests and changes of expression of the GDNF and MOR detected over time among groups were tested with one-way or two-way ANOVA, with repeated measures followed by Bonferroni post hoc tests, respectively. *p* < 0.05 was considered to be statistically significant.

## 3. Results

### 3.1. Establishment of Morphine Tolerance in Rats with Bone Cancer Pain

**Figure 1:**
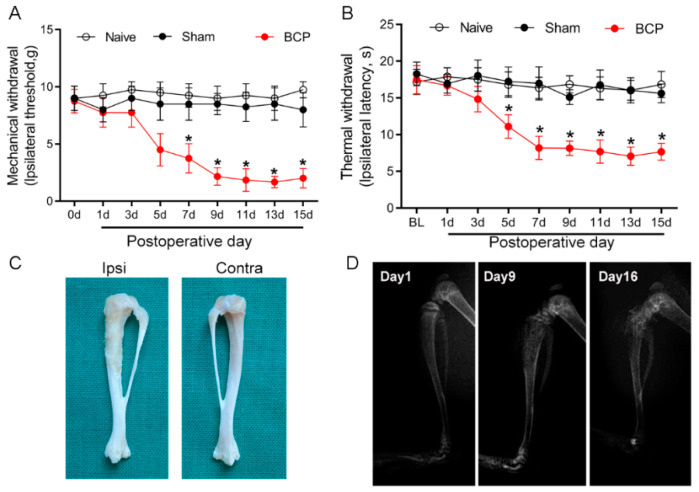
Walker 256 carcinoma cell intra-tibia injection induced bone destruction and bone cancer pain. The mechanical (**A**) and thermal (**B**) withdrawal latency decreased from day 5 after tumor cell inoculation, and hyperalgesia lasted from day 9 to day 15. Sham-operated (heat killed tumor cells) or naïve rats did not present an alteration in pain threshold compared with baseline. BCP, bone cancer pain. *n =* 8 rats. * *p* < 0.05, significantly different from both naïve and sham groups. (**C**) The ipsilateral tibia bone showed erosion and destruction of the medullary bone 15 days after intratibial injection of the carcinoma cells. (**D**) A representative X-ray image showed bone destruction in the distal part of the tibia after cancer cell injection.

Carcinoma cell inoculation induced local tumor proliferation and bone destruction, and resulted in BCP. The von Frey test and Hargreaves test were employed to evaluate the development of mechanical and thermal hyperalgesia in BCP rats. The baseline of mechanical and thermal withdrawal latencies in all of the tested rats was not significantly different among the groups (*p* > 0.05) (Figure 1A,B). Compared with the sham group, the paw withdrawal latency decreased from day 5 after tumor cell inoculation in the rats of the BCP group (*p* < 0.05), and hyperalgesia lasted from day 9 to day 15. The ipsilateral tibia bone showed erosion and destruction of the medullary bone 15 days after the intratibial injection of carcinoma cells (Figure 1C). In contrast, bone destruction was not observed in the contralateral tribals. The X-ray radiographic results showed a sign of bone destruction in the ipsilateral tibia on POD 9 and POD 16 (Figure 1D). These results indicate that the BCP model was established successfully.

**Figure 2:**
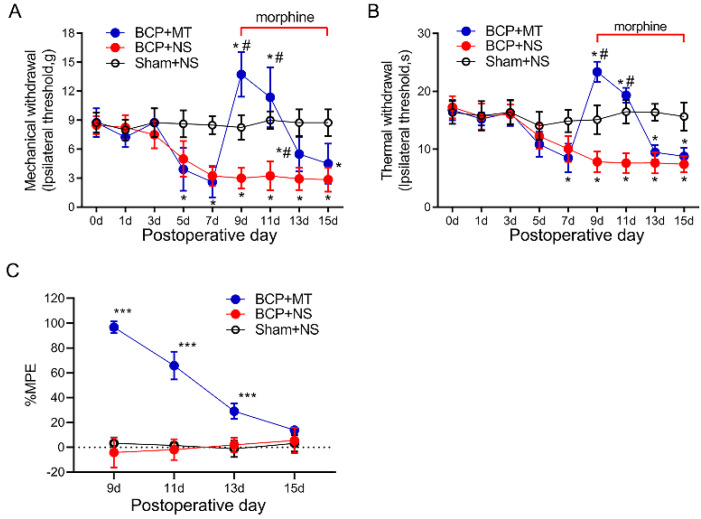
Chronic morphine administration induced analgesic tolerance in bone cancer pain rats. The mechanical (**A**) and thermal (**B**) withdrawal latency decreased and reached a stable state from day 9 after inoculation of the cancer cells. On day 9 after the tumor cell, paw withdrawal threshold after the first morphine injection was significantly increased in the rats with BCP. This increase was markedly attenuated and nearly diminished after 7 days of repeated injection. Intrathecal injection of saline did not affect the paw withdrawal latency both in the BCP and sham group. BCP+MT, morphine tolerance rats with BCP. BCP+NS, BCP rats treated with normal saline (NS). Sham+NS, sham operated rats treated with normal saline. *n* = 8 rats. * *p* < 0.05, significantly different from the sham+NS group. # *p* < 0.05, significantly different from the BCP+NS group. (**C**) The percentage of the maximum possible effect (%MPE) of morphine gradually decreased in the BCP+MT group. *n* = 8 rats. *** *p* < 0.001, significantly different from BCP+NS group.

As the PWTs and PWLs of rats reached a stable state from day 9 after the inoculation of cancer cells, we chose POD 9 as the starting time for the consecutive intrathecal injection of morphine. The tail-flick tests showed that the injection of morphine (10 μg i.t, twice a day, 8 h intervals) on POD 9 exhibited a potent analgesic efficacy compared with the BCP+NS and Sham+NS group (*p* < 0.05) (Figure 2A,B). The %MPE of rats in the BCP+MT group began to decrease on POD 11 and then decreased gradually (Figure 2C). On POD 15, the %MPE of the BCP+MT and BCP+NS groups was not significantly different, which indicated the successful establishment of MT in BCP rats.

### 3.2. Chronic Morphine Reduced the Level of GDNF in the Spinal Cord of BCP Rats

**Figure 3:**
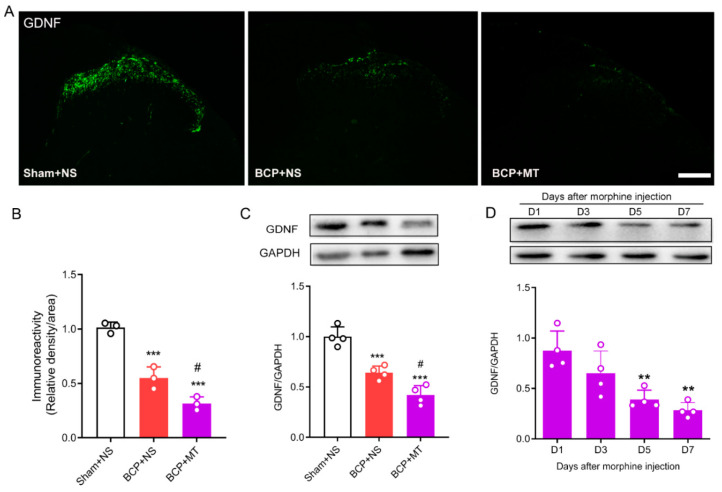
Chronic morphine reduced the level of GDNF in the spinal cord of BCP rats. (**A**,**B**) The immunochemistry test demonstrated that GDNF was located in the superficial laminae of the spinal cord. Compared with the sham operated rats treated with normal saline (sham+NS), the expression of GDNF was reduced in the BCP rats treated with normal saline (BCP+NS) and morphine (BCP+MT). *n* = 3 rats. *** *p* < 0.001, significantly different from sham +NS group. # *p* < 0.05, significantly different from the BCP+NS group. Scale bar = 200 μm. (**C**) Western blot demonstrated that expression of GDNF was decreased in BCP+MT rats compared with both the sham+NS and BCP+NS rats. *n* = 4 rats. *** *p* < 0.001, significantly different from the sham+NS group. # *p* < 0.05, significantly different from the BCP+NS group. (**D**) Western blot demonstrated that expression of GDNF was time-dependently reduced after chronic morphine treatment. *n* = 4 rats. ** *p* < 0.01, significantly different from the expression on Day 1.

To determine whether chronic morphine exposure alters the level of GDNF, we evaluated the expression of GDNF in the lumbar spinal cord by immunofluorescence staining and Western blot tests. The immunochemistry test demonstrated that GDNF was located in the superficial laminae of the spinal dorsal horn (Figure 3A). Nine days after the inoculation of tumor cells, we found that the expression of GDNF in the BCP group was significantly decreased compared with that in the Sham group. Furthermore, after 7 days of consecutive injections of morphine, the expression of GDNF was significantly downregulated compared with the BCP+NS group (Figure 3A,B). In line with the immunochemistry results, the Western blots showed that the expression of GDNF in the spinal cord decreased after the establishment of BCP, and GDNF was further downregulated after chronic morphine administration in BCP rats (Figure 3C). In addition, we further discovered that the level of GDNF was reduced significantly in a time-dependent manner after consecutive treatment of morphine (Figure 3D).

### 3.3. Intrathecal Injections of Lentiviral Vector Mediated GDNF Induce Persistent and Local Gene Expressions in Spinal Cord

**Figure 4:**
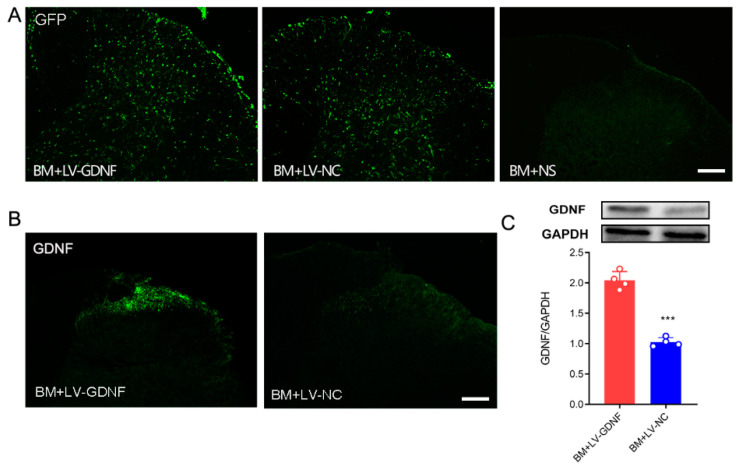
LV-GDNF intrathecal injection upregulated GDNF in the spinal cord of MT rats with BCP. (**A**) GFP expression was detected in the spinal cord of bone cancer pain and morphine tolerance rats treated with lentivirus-mediated GDNF (BM+LV-GDNF) and lentivirus of negative control (BM+LV-NC), but not in the BM rats treated with normal saline (BM+NS). Scale bar = 200 μm. (**B,C**) The overexpression of GDNF was detected by the immunochemistry test and Western blot in the spinal cord of the BM+LV-GDNF rats. *n* = 4 rats. *** *p* < 0.001, significantly different from the BM+LV-GDNF group.

To further clarify the influence of GDNF on MT in BCP rats, we constructed a recombinant lentivirus vector expressing GDNF. In BM rats that received intrathecal injection of lentivirus, the expression of GFP was observed both in the gray and white matter of the spinal cord (Figure 4A). Both immunochemistry and Western blot tests showed that in BM rats that received LV-GDNF, the protein level of GDNF in the lumbar spinal cord was up-regulated significantly compared with the rats that received negative control lentivirus (Figure 4B,C).

### 3.4. Upregulation of GDNF Alleviated the Morphine Tolerance in Bone Cancer Pain Rats

**Figure 5:**
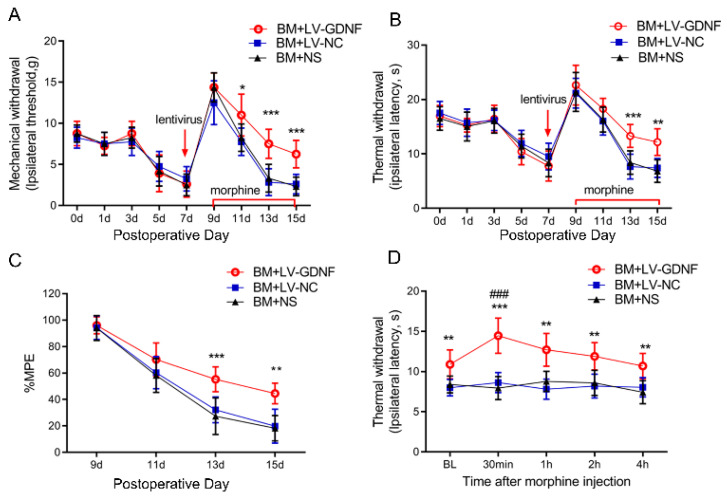
Upregulation of GDNF alleviated morphine tolerance in bone cancer pain rats. The mechanical (**A**) and thermal (**B**) withdrawal threshold indicated that LV-GDNF injection alleviated the morphine analgesic tolerance in BCP rats. *n* = 8 rats. * *p* < 0.05, ** *p* < 0.01, *** *p* < 0.001, significantly different from bone cancer pain and morphine tolerance rats treated with normal saline (BM+NS). (**C**) The percentage of the maximum possible effect (%MPE) of morphine was detected by the tail-flick latency test in the bone cancer pain rats. *n* = 8 rats. ** *p* < 0.01, *** *p* < 0.001, significantly different from BM rats treated with normal saline (BM+NS). (**D**) Time course of morphine analgesic effect in BM rats was detected by the thermal paw withdrawal latency on POD 15. *n* = 8 rats. ** *p* < 0.01, *** *p* < 0.001, significantly different from BM+NS group. ###, *p* < 0.001, compared with the corresponding baseline.

We further examined the effect of the intrathecal administration of LV-GDNF on the behavioral responses to morphine exposure in BCP rats. GDNF-GFP-Expressing or negative control lentivirus was intrathecally injected in rats on POD 7 before chronic morphine injection. The nociceptive behavior changes of rats were evaluated by PWT, PWL, and MPE%. Before the lentivirus was injected, there were no significant differences in the pain threshold of the rats among the three groups after the implantation of tumor cells (Figure 5A,B). The first injection of morphine (POD 9) significantly suppressed the mechanical and thermal hyperalgesia in BCP rats. However, the antinociception of morphine was obviously reduced and even diminished following 7 consecutive days of administration in rats treated with LV-NC or saline. Conversely, the intrathecal injection of LV-GDNF alleviated the MT in BCP rats (Figure 5A,B). The MPE% of morphine in BCP rats injected with the LV-NC or saline time-dependently decreased on days 5 and 7 after continuous morphine infusion. However, for the BCP rats with LV-GDNF, the MPE% of morphine was relatively higher compared with LV-NC treated rats on day 5 and 7 (Figure 5C).

To explore whether the effect of LV-GDNF was a direct antinociceptive action or due to its effect to attenuate MT in BCP rats, we measured the PWL before or after the last injection of morphine (POD 15). We found that morphine significantly increased the PWL in the LV-GDNF group, and the analgesic effects of morphine administration peaked at 30 min and were sustained for about 4 h. Meanwhile, the PWL in BM rats receiving LV-NC or saline remained unchanged after the intrathecal injection of morphine on POD 15. Given that the PWL change characteristics in BM rats injected with LV-GDNF were consistent with the pharmacodynamic efficacy of morphine, these results suggest that the overexpression of GDNF could restore sensitivity to morphine to exert an analgesic effect in BCP rats (Figure 5D).

### 3.5. Changes of μ-Opioid Receptor (MOR) in the Spinal Cord after LV-GDNF Injection

**Figure 6:**
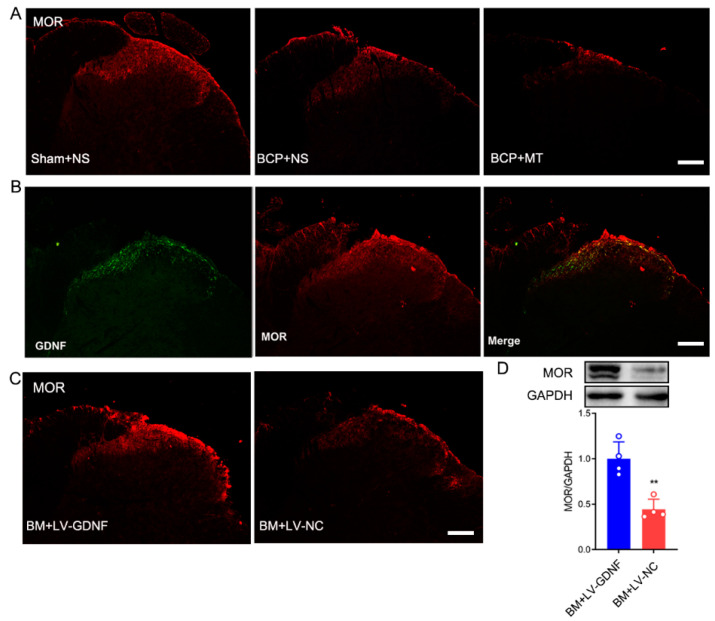
Upregulation of GDNF recovered MOR expression in morphine tolerance rats with bone cancer pain. (**A**) MOR expression was reduced in the spinal cord of bone cancer pain rats treated with normal saline (BCP+NS), and further downregulated in BCP rats with morphine tolerance (BCP+MT). Scale bar = 200 μm. (**B**) The double immunochemistry test demonstrated that GDNF and MOR were coexpressed in the spinal cord of rats. Scale bar = 200 μm. (**C**,**D**) The expression of MOR in the spinal cord of morphine tolerance rats with bone cancer pain was restored by LV-GDNF injection, but not LV-NC intrathecal injection. *n* = 4 rats. ** *p* < 0.01, significantly different from the LV-NC group.

Given that opioids exert nearly all their clinically relevant actions through stimulation of MOR, we explored the expression of MOR after the development of MT in BCP rats and evaluated the spatial relationship between GDNF and them in the spinal cord. The immunochemistry results showed that after BCP induction, the expression of MOR was downregulated in the spinal cord of BCP rats (Figure 6A). After 7 days of consecutive injections of morphine, the expression level of MOR in the BCP+MT group was further downregulated compared with the BCP+NS group. The double immunochemistry test showed that GDNF was coexpressed with MOR in the dorsal horn of the spinal cord (Figure 6B). Thus, we investigated whether the overexpression of GDNF affected the expression of MOR in the spinal cord. Interestingly, we discovered that upregulated GDNF in the spinal cord of BM rats resulted in the density and protein expression of MOR showing an obvious up-regulation. Conversely, there was no change in the level of MOR expression in the lumbar spinal cord in the BM rats injected with LV-NC (Figure 6C,D). Thus, the role of GDNF could modify the behavioral responses to chronic morphine exposure, which may be related to its regulating action on MOR.

## 4. Discussion

In the present study, we found that upregulating the expression of GDNF by intrathecal injection lentivirus vector expressing GDNF could alleviate MT in the BCP rats. Many attempts have been made to prevent MT, but, as yet, none have fully succeeded [16]. Gene therapy with lentivirus is a powerful method to deliver molecules to the nervous system in a site-specific manner. Lentiviral vector is one of the ideal gene delivery tools as it mediates long-term and stable expression patterns, inducing limited inflammation, and has a relatively large cloning capacity. A large body of evidence shows that targeted delivery of exogenous GDNF to the specific brain or spinal regions by viral vectors may be exploited as a potential therapeutic strategy in the treatment of Parkinson’s disease and neuropathic pain [17,18,19], suggesting that using lentivirus inducing a local up-regulation of GDNF is likely to be a potential therapy. Our study demonstrates that the GDNF level was decreased after applying morphine. It has been suggested that low levels of GDNF may increase sensitivity to abused drugs because GDNF protects DAergic neurons from drug-induced plasticity [11,20]. Therefore, we constructed a recombinant lentiviral vector expressing GDNF and injected it into the spinal cord to examine the behavioral and biochemical changes of rats. It has been reported that the expression of GFP or the target gene was founded in the spinal cord at day 3 or day 4 after lentivirus intrathecal injection, and significantly increased from then to day 7, and the sustained expression lasted more than 2 weeks [21,22]. Some studies even reported that the expression persisted longer than 9 months after lentivirus injection [23]. In the present study, the lentivirus was intrathecally injected on POD 7 after tumor cell inoculation, and the spinal cord was harvested on POD 15 to observe the expression of GDNF. As shown in Figure 4, the expression of GDNF in spinal cord was up-regulated by lentivirus in BCP rats on POD 15, which indicated that the effect of LV-GDNF on GDNF expression lasted at least 9 days. We found that LV-GDNF was successfully transduced into the spinal cord, and it could improve the analgesic effect of morphine and alleviate the development of tolerance to morphine.

In this study, to accurately mimicking clinical scenarios, we established an MT model on the base of BCP. It is well known that opioids are mainly used for the treatment of moderate to severe pain, and patients usually do not consume opioids when they are not in a pain condition. However, many studies on opioid tolerance often use pain-free rats to induce MT models, which is not fully in conformity with the clinical situations [24]. For this reason, we explored the development of opioid tolerance in animals with BCP, a model that includes prolonged moderate to severe pain.

GDNF has been reported to participated in the maintenance and development of striatal dopaminergic neurons [10,25] and regulates dopamine transmission [26], which is considered to play a crucial role in the addictive effects of opioid abuse. Further studies indicate that GDNF could be involved in the development of opioid addiction, as chronic cocaine decreased the levels of GDNF in the striatum [27], and GDNF blocks the biochemical and behavioral responses to repeat opioid administration [28,29]. The mechanism through which GDNF reverses the behavioral responses to drug abuse remains unknown. In our present study, we found that the levels of GDNF in the spinal cord were decreased in a time-dependent manner after the repeated administration of morphine, indicating that GDNF may be involved in the development of MT. Next, we showed that overexpressing GDNF by intrathecal injection of LV-GDNF was effective at improving morphine-induced behavioral disorders. Our results show that GDNF could delay morphine analgesic tolerance and alleviate bone cancer-induced mechanical and thermal hyperalgesia in rats. Interestingly, the effect of LV-GDNF on morphine analgesia in BCP rats was temporally changed. As shown in Figure 2 and Figure 5, morphine produced a robust analgesic effect in BCP rats at the first use on POD9, whereas LV-GDNF did not showed detectable effect. However, repeated administration of morphine induced obviously antinociceptive tolerance, and LV-GDNF relieved the morphine tolerance in BCP rats. Even though LV-GDNF partial restored the pharmacodynamic characteristics of morphine, the analgesic effect decreased gradually. Taken together, these results indicate that LV-GDNF attenuated morphine tolerance, but did not completely prevent it.

Opioid tolerance limits its clinical use for treating cancer pain. However, its definite molecular mechanism is still unclear. Given that MOR is the most important opioid receptor responsible for the analgesic effect of morphine, numerous studies have focused on the MOR density and function adaptive changes underlying opioid tolerance and dependence [30,31]. However, studies on the regulation of MOR after chronic opioid treatment have given conflicting results, depending on the experimental conditions [32,33,34]. Our present results showed that the expression of MOR was decreased in the spinal cords of BCP rats, and such a decrease was much more obvious in the BM group. The finding was consistent with a previous study that sarcoma cell injection down-regulated MOR expression in the spinal cord [35]. Interestingly, we were surprised to find that the level of MOR in the spinal cord was up-regulated after intrathecal injection with LV-GDNF. Furthermore, the overexpression of GDNF attenuated the development of MT in the BCP rats. Therefore, we tentatively put forward that one possible mechanism of GDNF alleviating morphine analgesic tolerance as that it up-regulates the expression MOR. Certainly, further studies need to be done to identify the precise molecular steps through which GDNF increases the density of MOR to reverse the development of tolerance to morphine.

## 5. Conclusions

In conclusion, the current study demonstrates that the down-regulation of GDNF is involved in the development of tolerance to morphine, and overexpressing GDNF in the spinal cord could attenuate the decreasing MPE% of morphine in BCP rats that received chronic morphine administration. This may be due to the role of GDNF to restore the density of MOR in the spinal cord of BCP rats. Thus, GDNF may be a potential target for preventing the development of morphine tolerance during BCP treatment.

## Data Availability

Data in the current study are available from the corresponding author upon request.
